# Bacterial Communities in the Rhizospheres of Three Mangrove Tree Species from Beilun Estuary, China

**DOI:** 10.1371/journal.pone.0164082

**Published:** 2016-10-03

**Authors:** Peng Wu, Xiaofei Xiong, Zhanzhou Xu, Chuqian Lu, Hao Cheng, Xiangli Lyu, Jinghuai Zhang, Wei He, Wei Deng, Yihua Lyu, Quansheng Lou, Yiguo Hong, Hongda Fang

**Affiliations:** 1 South China Sea Environmental Monitoring Center, State Oceanic Administration, Guangzhou, China; 2 State Key Laboratory of Tropical Oceanography, South China Sea Institute of Oceanology, Chinese Academy of Sciences, Guangzhou, China; Chinese Research Academy of Environmental Sciences, CHINA

## Abstract

The bacterial communities played important roles in the high productivity mangrove ecosystem. In this study, we investigated the vertical distributions of rhizosphere bacteria from three mangrove species (*Bruguiera gymnorrhiza*, *Kandelia candel* and *Aegiceras corniculatum*) in Beilun Estuary, China using high throughput DNA pyrosequencing of the 16S rRNA gene. Phylogenetic analysis showed that bacterial communities from mangrove rhizosphere sediments were dominated by *Proteobacteria* (mostly *Deltaproteobacteri*a and *Gammaproteobacteria*), followed by *Chloroflexi*, *Bacteroidetes*, *Planctomycetes* and *Acidobacteria*. However, the ANOVA analysis on Shannon and Chao1 indices indicated that bacterial communities among sediments of the three mangrove species varied more strongly than the sampling depths. In addition, the PCA result demonstrated that the bacterial communities could be separated into three groups according to the mangrove species. Moreover, the dominated orders *Rhodospirillales*, GCA004 and envOPS12 were significantly different among sediments of the three mangrove species. The results of this study provided valuable information about the distribution feature of rhizosphere bacteria from Chinese mangrove plants and shed insights into biogeochemical transformations driven by bacteria in rhizosphere sediments.

## Introduction

Mangroves are unique intertidal ecosystems in tropical and subtropical regions, where they play an essential roles in providing nursery habitats for aquatic animals, degrading contaminants and protecting the coast [1, 2]. Adapted to intertidal zones, they are subjected to highly variable physicochemical conditions of salinity, flooding, light, temperature and nutrient, which give rise to the high bacterial diversity that characterizes mangrove ecosystems [3, 4]. The diverse microbial communities can continuously transform nutrients from dead mangrove vegetation into sources of nitrogen, phosphorus and other nutrients which can be used by mangrove trees [5]. As a result, bacteria are important to the productivity, conservation and rehabilitation of mangrove ecosystems [5].

Mangrove trees can oxidize the sediment by supplying oxygen to the anaerobic subsediment through their aerial roots [6]. In addition, mangrove root exudates can serve as a nutrient source for bacteria [5]. These changes induced by the trees could influence the proliferation of certain groups of bacteria in the rhizosphere [5, 7]. In terrestrial environments, previous studies confirmed that bacterial communities in rhizosphere were influenced by plant species, and even that the rhizosphere with respect to plant species richness and community size [8–10]. However, in mangrove, bacterial compositions in rhizospheres of different tree species are not well known.

Most of the previous studies focused on the bacterial distribution from mangrove surface sediment in the horizontal direction [11–14]. In addition, seasonal changes of bacterial community in mangrove were also discussed in previous studies [14–16]. The vertical distribution of bacterial composition was fully investigated in terrestrial ecosystem. For example, Yu and Steinberger [17] found vertical changes of soil microbial community under the canopies of *Zygophyllumdumosum* and *Hammadascoparia*. Lee et al. [18] also showed that bacterial compositions from rhizosphere soils of a flooded rice paddy differed along a depth gradient and suggested that the oxygen concentration might be a determining factor. But, vertical profiles of bacterial structure in mangrove are still poorly understood.

The aim of the present study was to compare the vertical profiles of bacteria in the rhizospheres of three mangrove tree species (*Bruguiera gymnorrhiza*, *Kandelia candel* and *Aegiceras corniculatum*). In order to reduce possible anthropogenic effects, a pristine site in Beilun Estuary National Nature Reserve (a member of Man and Biosphere Programme, UNESO) was used as the study site, which constructed to protect mangrove located in Guangxi province, China. To thoroughly investigate the bacterial community, a barcoded pyrosequencing analysis of 16S rRNA gene was employed to understand bacterial communities in the mangrove rhizosphere from Beilun Estuary, China.

## Materials and Methods

### Ethics statement

Beilun Estuary National Nature Reserve approved this study development. The field studies did not involve endangered or protected species.

### Study area and sediment sampling

Sediment samples were collected on September 28, 2015 from Beilun Estuary National Nature Reserve, which was located in South China (21°31'00" N-21°37'30" N, 108°00'30" E-108°16'30" E) with an area of 3000 hm^2^. This reserve contained 15 species of mangrove trees belong to 11 families. In this study, three species of mangrove trees were investigated, including *Bruguiera gymnorrhiza* (Bru), *Kandelia candel* (Kan) and *Aegiceras corniculatum* (Aeg) which were the common and dominant species in this reserve. Three plants of each species (about 1.5 m tall) were selected within distance of 1 to 10 m from each other. For each individual plant, the rhizosphere sediments were sampled vertically along the base of the plant. Then, the rhizosphere sediments corresponding to 0 (surface), 10 and 20 cm depths were collected. Finally, samples of each species in triplicate from each depth were mixed to homogeneity to generate a representative composite sample for further analysis. Samples were kept in sterile plastic bags, maintained in an ice box for transporting to the laboratory, and stored at -20°C for DNA extraction.

### Total community DNA extraction and Illumina HiSeq sequencing

Total genomic DNA was extracted directly from 1.0 g of the sample using FastDNA^®^spin kit (MP bio, Santa Ana, USA) following the manufacturer’s protocol. The bacterial community was analyzed using Illumina HiSeq sequencing of 16S rRNA gene amplicons. PCR amplifications were conducted in triplicate with the primer set 515F (5’-GTGCCAGCAGCCGCGGTAA-3’) and 907R (5’-CCGTCAATTCCTTTGAGTTT-3’) that amplified the V4-V5 region of the 16S rRNA gene. The reverse primer contained a 6-bp error-correcting barcode unique to each sample. DNA was amplified following the protocol described previously [19]. Pyrosequencing was performed on the Illumina HiSeq platforms at Novogene Bioinformatics Technology Co., Ltd, Beijing, China.

Pairs of reads from the original DNA fragments were merged by using FLASH [20]. Sequencing reads were assigned to each sample according to the individual unique barcode. QIIME [21] and UPARSE [22] were used to analyze the sequencing reads and pick operational taxonomic units (OTUs). Sequences were assigned to OTUs at 97% similarity. For each OTU, a representative sequence was selected and used to assign taxonomic composition by using the RDP classifier [23]. In case the read numbers influence the statistical diversity indices, especially for the Chao1 and Shannon indices, the numbers of the bacterial reads in each sample were normalized to 43,632 reads. Finally, Shannon index, Chao1 index and Good’s coverage for the nine samples with the normalized sequencing reads were determined as described previously [24].

### Statistical analysis

The significant differences of bacterial composition were analyzed by one-way ANOVA using SPSS 22.0 software package (p<0.05). Principle component analysis (PCA) was applied to compare the bacterial communities among all the samples in R software (version 3.2.3). Moreover, the representative sequences of the most dominant OTUs (>20 sequences) were determined using ternary diagrams to find out the differences of bacterial community from sediments of the three mangrove species. All sequences obtained from this study were deposited in NCBI sequence read archive (SRA) under accession number SRP081285.

## Results

### Sequencing statistics and diversity estimates

A total of 556,274 qualified pyrosequencing reads were obtained from the nine rhizosphere samples. The numbers of qualified reads per sample ranged from 52,197 (Bru-0) to 68,687 (Kan-0) with a mean of 61,808 (Table 1). The rarefaction curves obtained with the normalized OTUs number nearly reached saturation level for all the samples, which demonstrated bacterial communities from the rhizospheres were well covered by pyrosequencing (S1 Fig). Using 3% sequence cutoff value, the OTUs number ranged from 4,589 to 5,011 across all samples, with *A*. *corniculatum* harboring the least number of OTUs among sediments of the three mangrove species (Table 1). The average of Shannon index of bacterial diversity from rhizosphere sediments of *B*. *gymnorrhiza* was higher than *K*. *candel* or *A*. *corniculatum* (Table 1). However, the Chao1 index varied from 5,179 to 5,811, with an average of 5,468 (Table 1). The coverage of each sample was almost similar (from 96.9% to 97.6%), which was consistent with the demonstration of the rarefaction curve (Table 1 and S1 Fig).

**Table 1:** Sequencing information and diversity estimates for rhizosphere samples collected from three mangrove tree species (*B*. *gymnorrhiza*, *K*. *candel* and *A*. *corniculatum*) obtained by pyrosequencing.

Samples	No. of qualified reads	OTUs	Shannon	Chao1	Coverage (%)
Bru-0	52197	4621	10.38	5413	97.6
Bru-10	66732	4953	10.34	5698	97.2
Bru-20	68097	4928	10.26	5587	97.1
Kan-0	68687	5011	10.33	5811	96.9
Kan-10	67031	4866	10.17	5534	97.1
Kan-20	63275	4609	10.17	5269	97.4
Aeg-0	54541	4589	10.21	5179	97.6
Aeg-10	55325	4632	10.23	5275	97.5
Aeg-20	60389	4733	10.24	5446	97.3

### Bacterial community structure from different rhizosphere sediments

**Fig 1:**
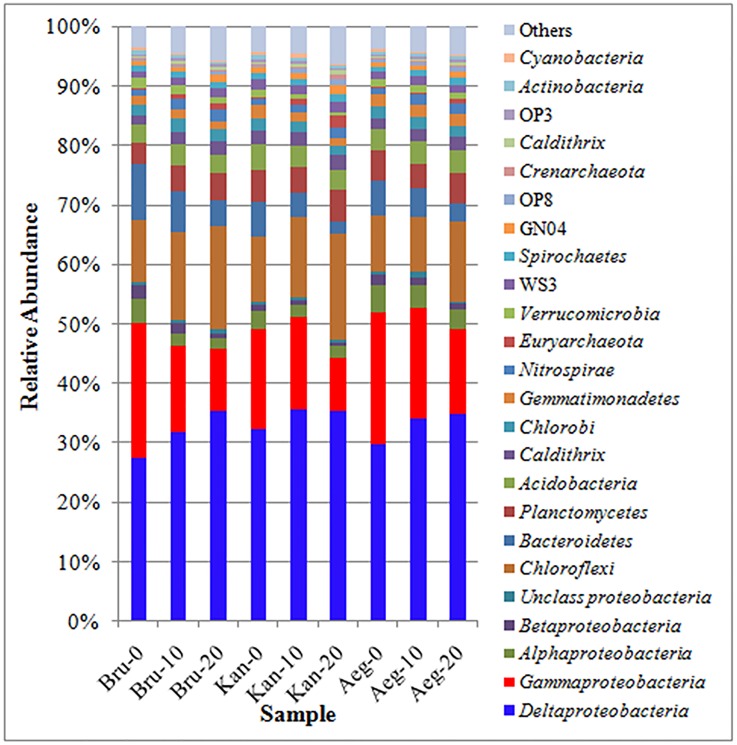
Relative abundance of the dominant bacterial phyla and proteobacterial classes identified through pyrosequencing targeting the 16S rRNA gene.

Relative abundance analysis showed that the top 20 phyla of bacteria from each sample accounted for over 90% of the total amplicons (Fig 1). *Proteobacteria* was the most dominant phylum covering 47.2–58.9% of the total amplicons which detected in all the nine samples. *Chloroflexi* was the second major phylum observed in this study, followed by *Bacteroidetes*, *Planctomycetes* and *Acidobacteria*. Interestingly, the relative abundance of *Chloroflexi* (9.1–17.2%) from each mangrove species increased with sampling depth, while *Bacteroidetes* (3.0–9.4%) decreased with sampling depth. However, the relative abundance of *Planctomycetes* (3.7–5.5%) and *Acidobacteria* (3.0–4.2%) showed a little difference between each sample (Fig 1). Besides, phyla *Euryarchaeota* and *Crenarchaeota* which belong to kingdom Archaea were found in all samples. In *Proteobacteria*, the *Deltaproteobacteri*a was the largest class (27.4–35.6% of all amplicons), followed by *Gammaproteobacteria* (8.9–22.6%) and *Alphaproteobacteria* (1.8–4.4%). Further, the relative abundance of *Gammaproteobacteria* apparently decreased with sampling depth. *Betaproteobacteria* (0.4–2.1%) and unclassified *Proteobacteria* (0.4–1.2%) were also found in different samples but they accounted for only small portions (Fig 1).

### The effect of mangrove species or sampling depth on bacterial community from rhizosphere sediment

**Fig 2:**
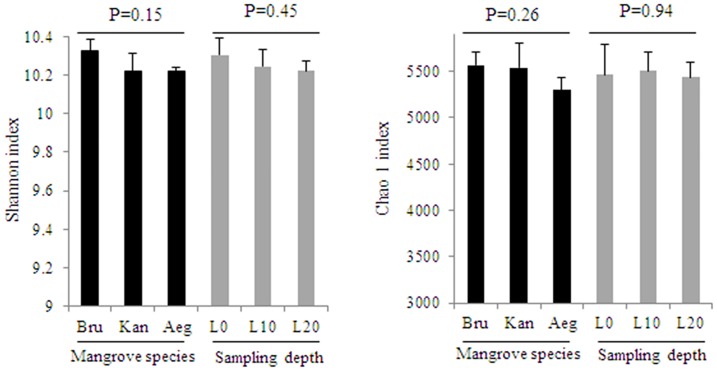
The analysis of statistical significant differences about Shannon and Chao1 indices for mangrove species and sampling depths (p<0.05).

**Fig 3:**
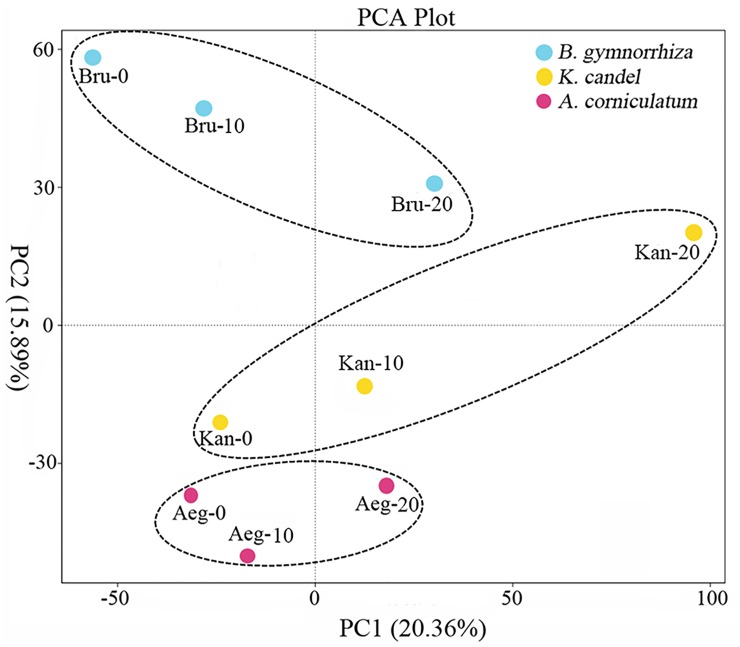
Principal coordinates analysis of bacterial communities from rhizosphere sediments.

One-way analysis of variance (ANOVA) analysis on Shannon and Chao1 indices showed bacterial communities among sediments of the three mangrove species varied more strongly than the sampling depths (Fig 2). Furthermore, to reduce the number of variables of the data and maintain as much variance as possible, PCA was used to compare bacterial communities between mangrove species and sampling depths. The PCA result also confirmed that the bacterial communities could be divided into three groups corresponding with the mangrove species. In contrast, the bacterial communities were not clearly differentiated along a depth gradient (Fig 3).

### Comparison of bacterial composition among sediments of the three mangrove species

**Fig 4:**
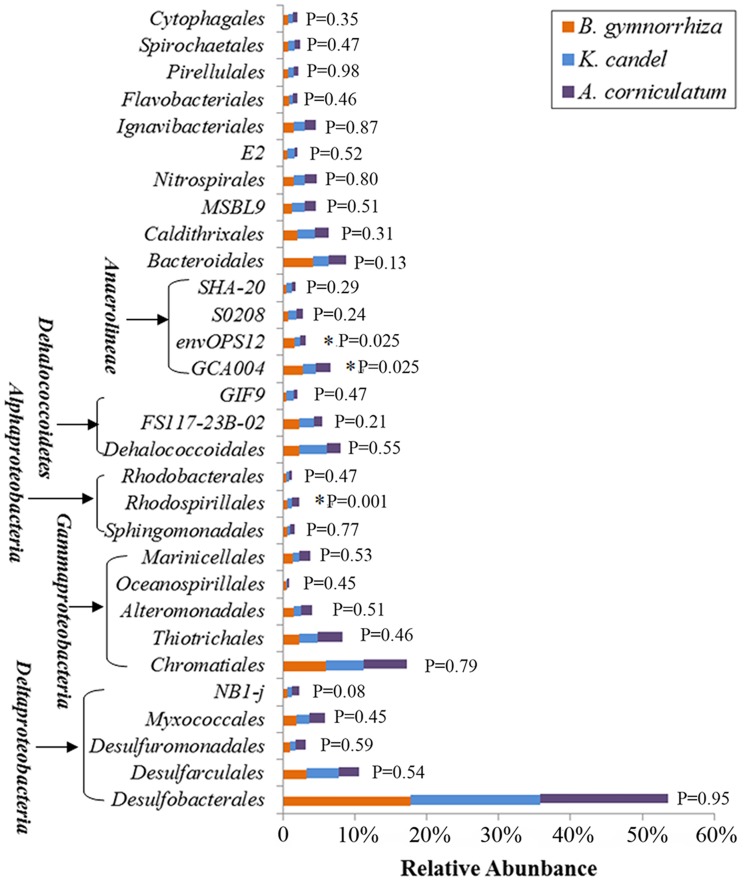
Relative abundance of the 30 most dominant bacterial groups on the order level. Symbols (*) represent significant differences among sediments of the three mangrove species (p<0.05).

The effect of mangrove tree species on bacterial distribution was further investigated in this study on the order level (Fig 4). Of these dominant orders, *Rhodospirillales*, and the candidate divisions (GCA004 and envOPS12) were significantly different among sediments of the three mangrove species (p<0.05). *Rhodospirillales* was highly abundant in rhizosphere sediment from *A*. *corniculatum*, while GCA004 and envOPS12 dominated in *B*. *gymnorrhiza*. In addition, *Bacteroidales* and NB1-j showed an obvious difference with the mangrove species (Fig 4). But, *Desulfobacterales* and *Chromatiales*, the first two dominant orders of all the investigated mangrove species, were almost invariable which accounted for 17.7–18.0% and 5.2–6.1% of total bacterial sequences, respectively (Fig 4).

**Fig 5:**
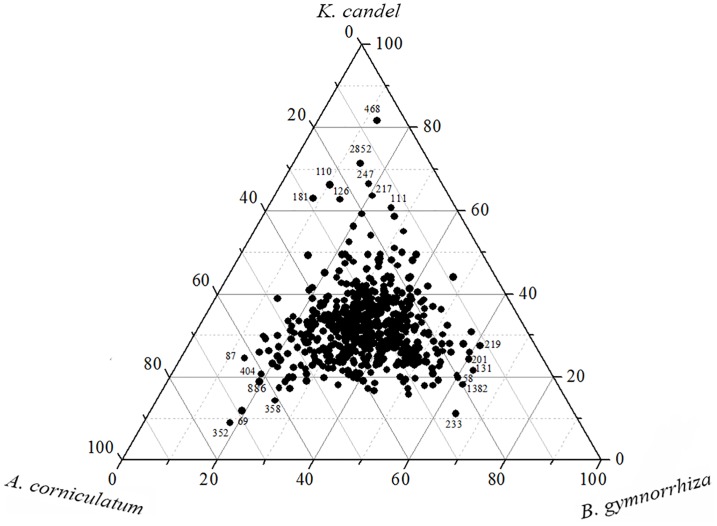
Ternary plots showing the ratios of the OTUs (≥20 reads) about sediments of the three mangrove species (*B*. *gymnorrhiza*, *K*. *candel* and *A*. *corniculatum*).

In term of OTUs (3% sequence cutoff value), Venn diagram were plotted to compare bacterial compositions from sediments of the three mangrove species. In rhizosphere sediments, 5,001 OTUs were shared by all the three mangrove species. On the other hand, 776 OTUs were only detected from the rhizosphere of *B*. *gymnorrhiza*. But, only 608 and 410 OTUs were only detected from the rhizospheres of *K*. *candel* and *A*. *corniculatum*, respectively (S2 Fig). The ternary plots of dominant OTUs revealed that different mangrove species contained special OTUs (Fig 5). Six OTUs (58, 131, 201, 219, 233 and 1382) were more prevalent in rhizosphere of *B*. *gymnorrhiza*. However, OTUs (110, 111, 126,181, 217, 247, 468 and 2852) were abundant in the rhizosphere of *K*. *candel*, while OTUs (69, 87, 352, 358, 404 and 886) dominated in *A*. *corniculatum* (Fig 5). From these OTUs, most were similar to 16S rRNA gene sequences reported from uncultured bacteria present in the sediment of marine, estuary or mangrove environment (Table 2). The percentage similarity of the analyzed OTUs with their closest blast hits ranged from 95% to 100%, respectively. What’s more, some OTUs from sediments of the three mangrove species were closely related to bacteria having the capacity to degrade organic pollutants (OTUs 219, 233, 352 and 886) or to recycle nutrients (OTUs 69, 111, 247, 358 and 1382) (Table 2).

**Table 2:** Taxonomic assignment of partial OTUs obtained from sediments of the three mangrove species and their closely related sequence identified using BLAST research.

OTU	Habitat	Database match with accession number in parentheses	Similarity	Origin or known traits
58	Bru	uncultured *Bacteroidetes* bacterium (GQ979660)	97%	an estuary surface sediment
131	Bru	Uncultured epsilon *Proteobacterium* (DQ394936)	99%	the sediment from Victoria Harbourin Hong Kong
201	Bru	Uncultured *Bacteroidetes* bacterium (AY822241)	95%	organically-enriched fish farm sediments
219	Bru	*Desulfatiferula berrensis* (NR133737)	98%	estuarine sediment/a n-alkene-degrading bacterium
233	Bru	Uncultured *Hydrogenophilaceae* bacterium (EU266782)	100%	a tar oil contaminant plume/anaerobic toluene degraders
1382	Bru	Uncultured *Cytophagales* bacterium (KC009968)	97%	the French Guiana coast/carbon dioxide fixing bacteria
110	Kan	Uncultured *Deltaproteobacterium* (DQ811827)	99%	the mangrove sediment
111	Kan	Uncultured *Deltaproteobacterium* (LC071293)	99%	the subsea floor off Hatsushima Island/anaerobic methane oxidization
126	Kan	Uncultured bacterium (FJ936722)	98%	volcano mud taken at Avachinsky (Kamtchatka)
181	Kan	Uncultured *Chloroflexi* bacterium (FJ902002)	98%	biomat in the sediment of cenote La Palita
217	Kan	uncultured archaeon (JX870284)	99%	the surface sediment of South China Sea
247	Kan	Uncultured *Chloroflexi* bacterium (LC070795)	95%	the subsea floor off Hatsushima Island/anaerobic methane oxidization
468	Kan	Uncultured *Gammaproteobacterium* (AB826802)	95%	the hadopelagic sediments in the Ogasawara Trench
2852	Kan	Uncultured *Deltaproteobacterium* (AB433126)	98%	deep subseafloor sediments at the Brazos-Trinity Basin, Mexico
69	Aeg	*Desulfatitalea tepidiphila* (AB719404)	98%	the tidal flat sediment/ a sulfate-reducing bacterium
87	Aeg	Uncultured bacterium (GQ246446)	99%	the North Yellow Sea sediments, China
352	Aeg	*Marinilabiliaceae* bacterium Q15 (KR809872)	100%	degrading different types of hydrocarbons
358	Aeg	*Malonomonas rubra* GraMal1 (NR026479)	99%	sulfur-reducing bacteria
404	Aeg	Uncultured *Deltaproteobacterium* (DQ112385)	99%	the intertidal mudflat sediment from Ganghwa Island, Korea
886	Aeg	*Desulfuromonas michiganensis* BB1 (NR114607)	98%	the freshwater sediment/tetrachloroethene-reducing anaerobic bacteria

## Discussion

In the present study, bacterial communities of different rhizosphere sediments from three mangrove tree species (*B*. *gymnorrhiza*, *K*. *candel* and *A*. *corniculatum*) were examined using high throughput DNA pyrosequencing of the 16S rRNA gene. However, bacterial composition from rhizosphere sediments mainly focused on the mangrove tree species, such as *Rhizophora mangle*, *Avicennia schaueriana*, *Laguncularia racemosa* and *Avicennia marina* [12, 15, 16, 25, 26]. Unfortunately, the discussion of bacteria from *B*. *gymnorrhiza*, *K*. *candel* or *A*. *Corniculatum* is lacking, while the three mangrove species are common in Guangxi Province of China.

### Bacterial community structures from rhizosphere sediments of *B*. *gymnorrhiza*, *K*. *candel* and *A*. *corniculatum*

In general, *proteobacteria* (mostly *Deltaproteobacteria* and *Gammaproteobacteri*a) was found to be the most abundant phylum in the rhizosphere sediment from *B*. *gymnorrhiza*, *K*. *candel* or *A*. *corniculatum* (Fig 1). Previous studies also showed that *Deltaproteobacteria* and *Gammaproteobacteri*a were dominated in rhizosphere of *A*. *schaueriana*, *L*. *racemosa* and *A*. *marina* [16, 25, 26]. Andreote et al. [27] also revealed the dominance of *Deltaproteobacteria* and *Gammaproteobacteria* from four distinct mangrove areas in Brazil. *Deltaproteobacteria* and *Gammaproteobacteria* were higher due to anaerobic condition of the mangrove sediment which drove selection of specific microbial groups such as sulfur-oxidizing bacteria [4, 28]. In this study, the high relative abundance of orders *Desulfobacterales* and *Chromatiales* from rhizosphere sediments belonging to sulfur-oxidizing bacteria dominated in classes *Deltaproteobacteria* and *Gammaproteobacteria*, respectively, which indicated mangrove plants played a key role in sulphur cycling (Fig 4). In the same, the two orders were reported to be prevalent from oil-contaminated soil of Brazilian pristine mangrove sediment [29].

Besides, *Chloroflexi* was the second dominant phylum in the three investigated mangrove species, which was not consistent with previous studies. Alzubaidy et al. [26] suggested that *Bacteroidetes* was the second largest phylum in *A*. *marina* from the Red Sea. However, second most abundant phylum from *A*. *marina* in Bay of Bengal was *Cyanobacteria*/*Chloroplast* in monsoon, whereas *Acidobacteria* in summer [16]. The phylum *Chloroflexi* was particularly widely distributed in many deep-sea sediments, representing up to 80% of the total bacterial 16S rRNA gene sequences at some sites [30]. Members of the phylum *Chloroflexi* were critical in the decomposition of organic matter [31]. This phylum can be divided into at least six major classes: *Chloroflexi*, *Dehalococcoidetes*, *Thermomicrobia*, *Caldilineae*, *Anaerolineae* and a clone cluster called SAR202 cluster [32]. The two classes (*Dehalococcoidetes* and *Anaerolineae*) were prevalent in this study (Fig 4). *Dehalococcoidetes* is strictly anaerobic and slow-growing which uses organohalide respiration via reductive dehalogenases (Rdh) as their sole mode of energy conservation [30]. *Anaerolinea*e appears to take key role in electron transfer to anodes; however, it is presently unclear whether they are directly involved or whether they produce metabolic intermediates from root exudates or soil organic matter, utilized subsequently by other directly anode-coupling microorganisms [33].

Furthermore, some dominated phyla involving in nitrogen cycle were found in mangrove rhizosphere sediments, such as *Planctomycetes*, *nitrospirae* and *Cyanobacteria* (Fig 1). Anammox bacteria belonging to phylum *Planctomycetes* had the unique metabolic ability to combine ammonium and nitrite or nitrate to form nitrogen gas under anoxic conditions [34, 35]. However, Phylum *nitrospirae* was one of the key players in the nitrogen cycle referring to nitrite oxidizing bacteria [36, 37]. In addition, phylum *Cyanobacteria* has been proved to contribute to nitrogen fixation in mangrove by many previous studies [38, 39].

### The influence of mangrove species on bacterial composition from rhizosphere sediments

The ANOVA analysis on Shannon and Chao1 indices and the PCA result indicated that bacterial communities from rhizosphere sediments were influenced much more by mangrove species than sampling depths (Figs 2 and 3). To compare with bulk sediment, the aboveground mangrove vegetation showed an important role in shaping rhizosphere bacterial community [13, 26, 40]. Besides, Gomes et al. [25] assessed bacterial compositions between the rhizospheres of two mangrove tree species and suggested that *A*. *schaueriana* and *L*. *racemosa* roots appeared to be able to impose a selective force on the bacterial communities from mangrove sediments and this phenomenon appeared to be plant species specific. Therefore, the significant influence of mangrove species on the rhizosphere bacterial community was further confirm in this study.

The differences of bacterial composition from the three investigated mangrove species showed that the dominant orders *Rhodospirillales*, GCA004 and envOPS12 were significantly different between each mangrove species (Fig 4). Members of *Rhodospirillales* belonged to a kind of photosynthetic anoxygenic bacteria which contained bacteriochlorophyll a as their major pigment and can use light to grow [4]. Basak et al. [14] also found *Rhodospirillales* was abundant in mangrove sediments of Sundarbans among the class *Alphaproteobacteria*. The predominant photosynthetic bacteria in anaerobic environments may contribute to the productivity of the mangrove ecosystems [41]. Orders GCA004 and envOPS12 belonged to class *Anaerolineae* which was found in a wide range of environments, including arctic permafrost, marine and freshwater sediments, sponges, the mammalian gastrointestinal tract and anaerobic sludge bioreactors [42]. The three orders *Rhodospirillales*, GCA004 and envOPS12 were all anaerobic bacteria, which may be related to the feature of mangrove sediment that was composed of thick organic matter and was anaerobic except for the surface sediment.

The ternary plots of dominant OTUs also confirmed that special OTUs associated with different mangrove species (Fig 5). Furthermore, most of the special OTUs from sediments of the three mangrove species were similar to uncultured bacteria, and some OTUs were closely related to bacteria with the ability of degrading organic pollutants or recycling nutrients (Table 2). These results demonstrated that rhizosphere bacteria in mangrove were highly diverse and made an essential contribution to the productivity of the mangrove ecosystem.

In conclusion, we provided the first insights into the vertical distribution of rhizosphere bacteria from three mangrove species in Beilun Estuary, China. Results indicated that the influence of mangrove tree species on the distribution of rhizosphere bacterial community was more strongly than sampling depths. Further studies are necessary to investigate whether any environmental factors can influence the bacterial community in mangrove. However, the bacterial community from each mangrove species in this study showed potential important ecological functions in mangrove ecosystems.

## Supporting Information

S1 Fig Rarefaction curves of the normalized OTUs number at 97% similarity. (TIF)

S2 Fig Venn diagram of bacterial composition from sediments of the three mangrove species. (TIF)
