# Supplementary figures and images for: Bacterial Communities in the Rhizospheres of Three Mangrove Tree Species from Beilun Estuary, China
Source: PLoS One. 2016 Oct 3;11(10):e0164082. doi: 10.1371/journal.pone.0164082 (PMC5047532; doi:10.1371/journal.pone.0164082)

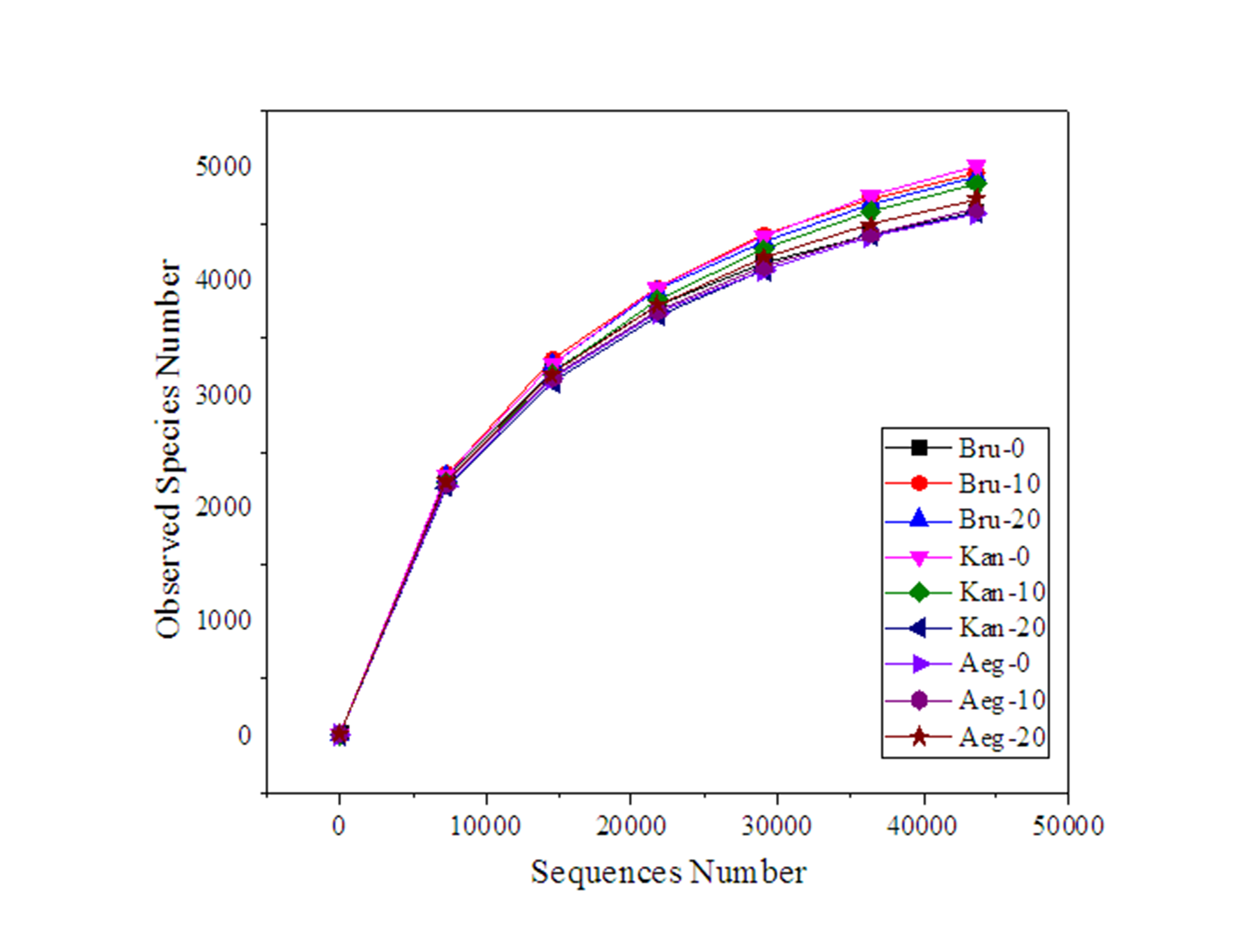

Supplement: S1 Fig — (TIF) [file pone.0164082.s001.tif]

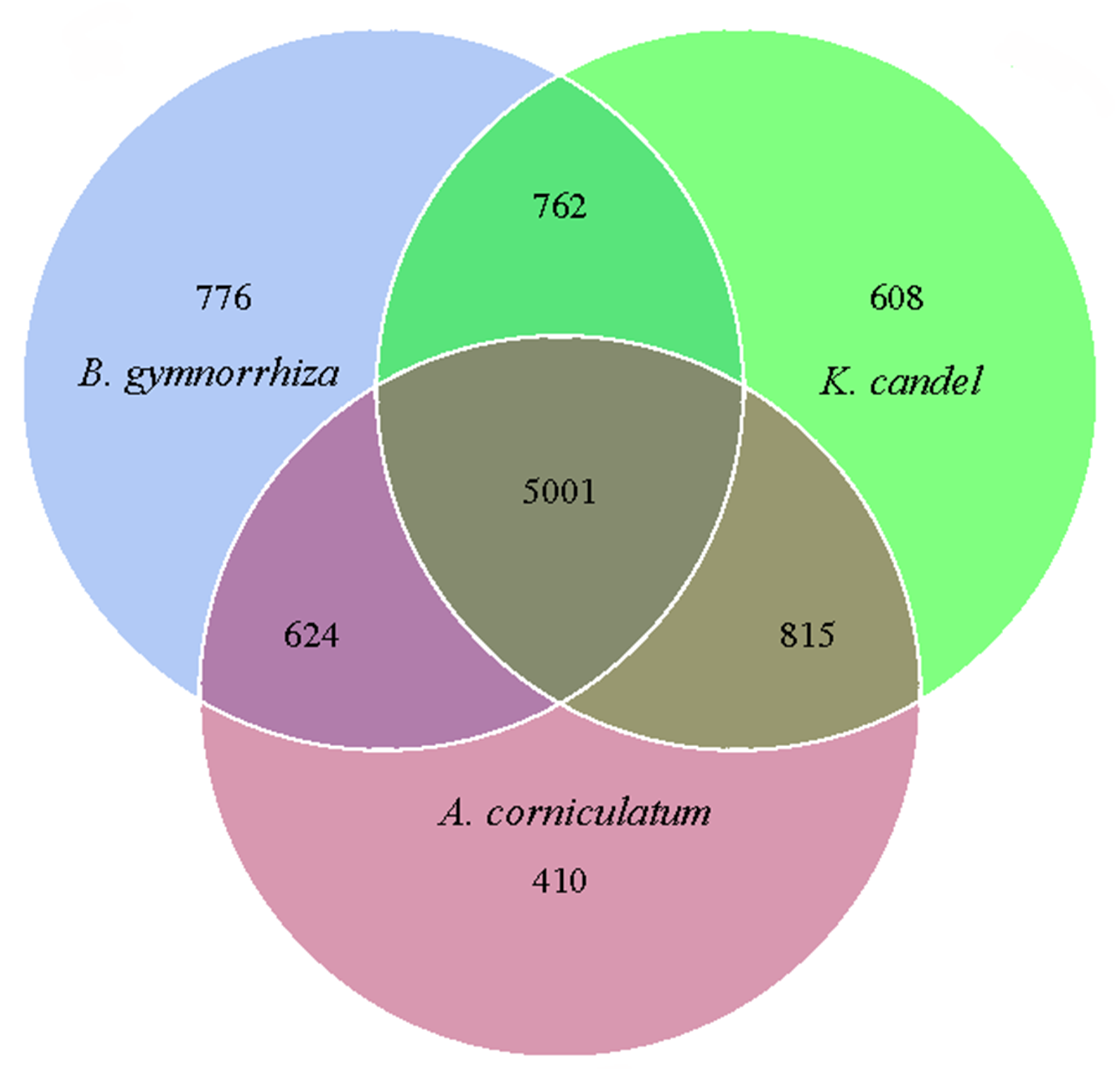

Supplement: S2 Fig — (TIF) [file pone.0164082.s002.tif]
